# Impact of Right Atrial Plication on Pulmonary Function and Heart Failure Symptoms: A Case Report

**DOI:** 10.7759/cureus.76447

**Published:** 2024-12-26

**Authors:** Shunya Ono, Kazuki Morooka, Retsu Tateishi, Kosaku Nishigawa, Takeyuki Kanemura

**Affiliations:** 1 Cardiovascular Surgery, IMS Katsushika Heart Center, Tokyo, JPN

**Keywords:** heart failure, pulmonary function, right atrial enlargement, right atrial plication, tricuspid regurgitation

## Abstract

An 85-year-old woman with long-standing atrial fibrillation and severe tricuspid regurgitation presented with worsening symptoms and massive right atrial enlargement (RAE). The patient experienced shortness of breath even during minimal exertion, such as walking within her house, which significantly impacted her daily activities. Surgical intervention, including tricuspid valve replacement and right atrial plication (RAP), led to significant symptomatic relief and improved pulmonary function. Postoperatively, the patient’s heart size and lung capacity showed significant recovery. This case underscores the potential benefits of the RAP in alleviating respiratory restriction due to lung compression caused by the enlarged right atrium and enhancing the quality of life of patients with severe RAE.

## Introduction

Long-standing atrial fibrillation (AF) is a known cause of biatrial enlargement and has been reported to increase the risk of worsening heart failure (HF) and arrhythmic death [1,2]. Although there are reports of improved respiratory function after left atrial plication (LAP), studies on the outcomes of right atrial plication (RAP) are scarce [3,4].

Herein, we present a case of severe tricuspid regurgitation (TR) accompanied by massive right atrial enlargement (RAE) that was successfully managed through surgical intervention, including tricuspid valve replacement (TVR) and RAP, leading to a significant improvement in respiratory function and alleviation of HF symptoms.

## Case presentation

An 85-year-old woman with a long history of chronic AF had been receiving medical therapy at another hospital for severe TR and prominent cardiomegaly. Otherwise, no significant past medical history, comorbidities, or notable demographic factors were identified. Shortness of breath had been present for several years but had progressively worsened over the past few months, with symptoms classified as New York Heart Association (NYHA) class III. This included breathlessness even during minimal activities, such as walking inside her home. Signs suggesting right-sided HF, such as leg swelling or jugular venous distension, were not observed. The reduction in lung volume due to the significant RAE was considered a major contributor to the symptoms. These progressive symptoms led to her referral to our hospital for surgical evaluation.

**Figure 1:**
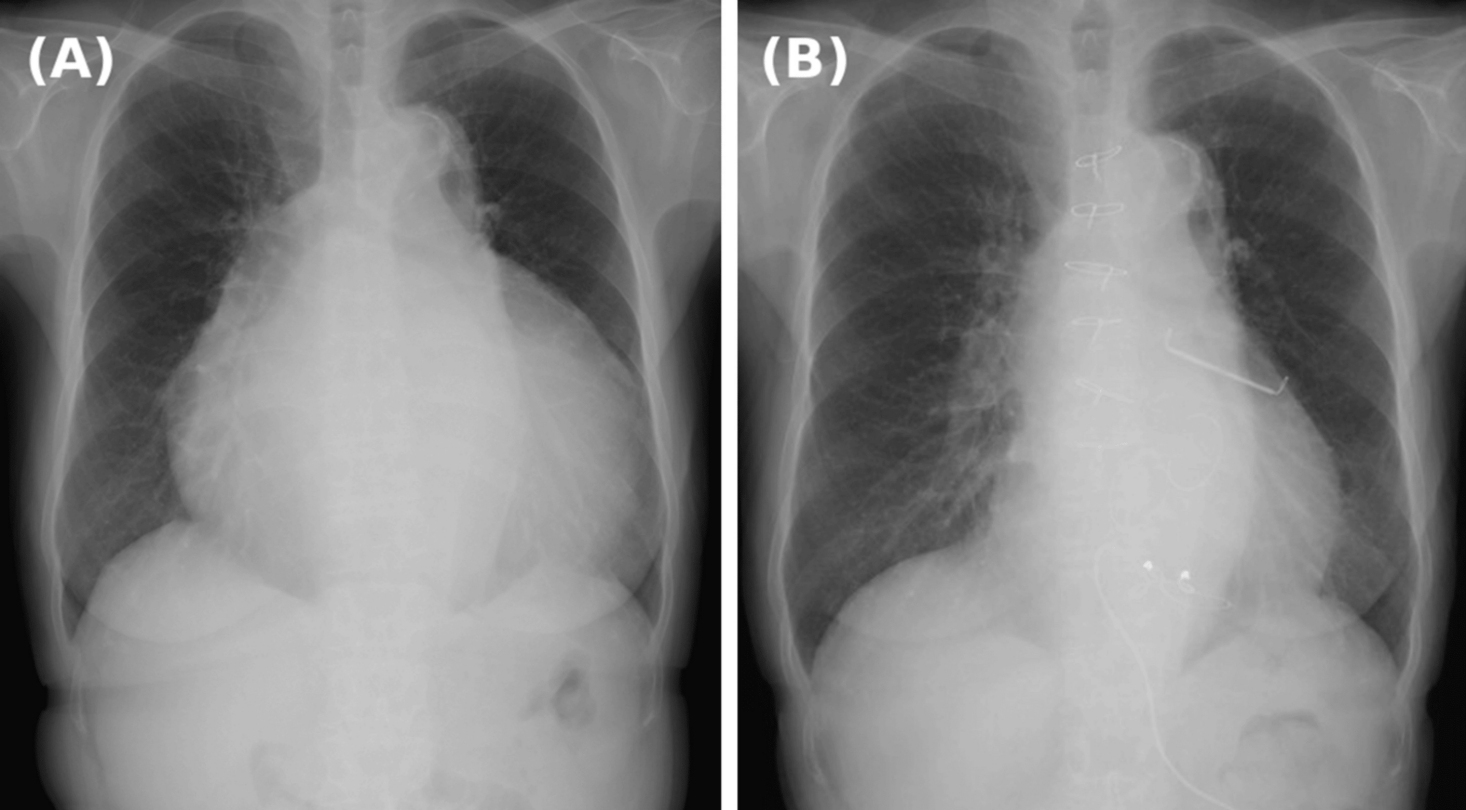
CXR findings (A) Preoperative CXR shows cardiomegaly with a CTR of 80%. (B) Postoperative CXR shows a significant reduction in CTR to 52%. CXR: chest X-ray; CTR: cardiothoracic ratio

**Figure 2:**
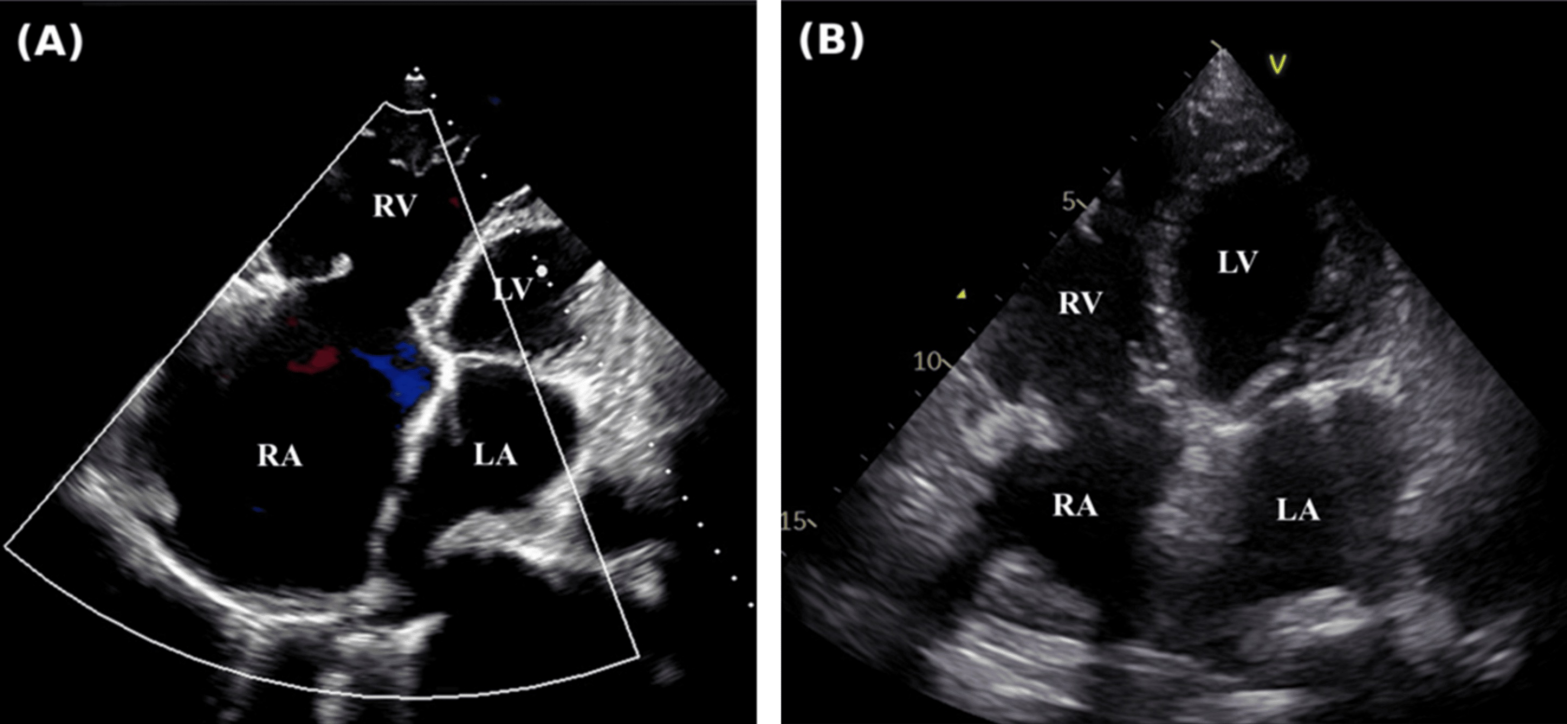
Echocardiography in a four-chamber view (A) Preoperative echocardiography shows severe RA enlargement. (B) Postoperative echocardiography shows normalization of RA size. LA: left atrium; LV: left ventricle; RA: right atrium; RV: right ventricle

CXR revealed significant cardiomegaly with a cardiothoracic ratio (CTR) of 80% (normal range: 42-50%) (Figure 1A). Electrocardiography revealed AF with a low fibrillatory wave amplitude. Echocardiography revealed a good ejection fraction of 62%; however, severe TR was caused by coaptation failure due to significant RAE, with a right atrial size of 114 × 82 mm and a tricuspid annular plane systolic excursion (TAPSE) of 14.6 mm, indicating mildly reduced right ventricular (RV) function (Figure 2A). In addition, the patient had moderate mitral regurgitation (MR) due to annular dilation. No congenital anatomical abnormalities were observed. Pulmonary function testing (PFT) showed a vital capacity (VC) of 1.1 L (53%), indicating restrictive ventilatory impairment, likely due to reduced lung volume caused by the enlarged right atrium (RA).

These findings, combined with the patient’s progressive symptoms, confirmed the diagnosis of severe TR with massive RAE requiring surgical intervention.

The surgery was performed via a median sternotomy, which was selected over minimally invasive approaches to ensure a more reliable technique for hemostasis and to minimize operative time. Cardiopulmonary bypass (CPB) was established with aortic cannulation and bicaval venous cannulation. The superior vena cava and inferior vena cava (IVC) were snared, and the RA was incised, extending from the cannulation site of the IVC toward the roof of the left atrium (LA). In the transseptal approach, the atrial septum was incised at the fossa ovalis and extended toward the roof of the LA to expose the mitral valve. The mitral valve showed no leaflet abnormalities; however, the regurgitation was attributed to annular dilation. Therefore, the ring annuloplasty using a 30 mm Physio Flex ring (Edwards Lifesciences, Irvine, California, USA) was performed. The atrial septum was closed using continuous 4-0 polypropylene sutures, incorporating a plication of the septum.

**Figure 3:**
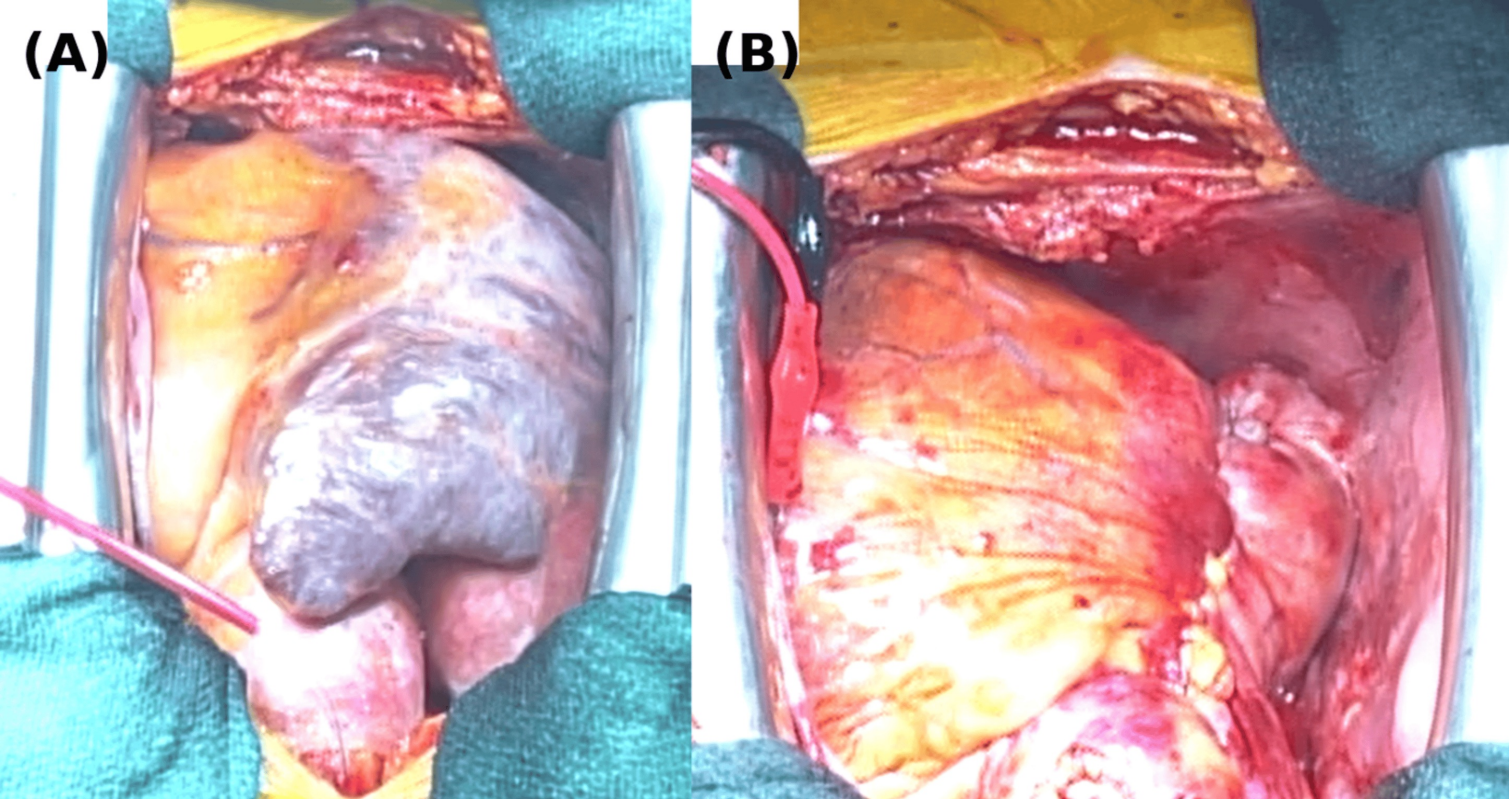
Intraoperative findings (A) The dilated right atrium before intervention. (B) Significant reduction in right atrial volume after intervention. The images are oriented with the patient’s head at the bottom and the foot at the top. The right side corresponds to the surgeon’s side, and the left side corresponds to the patient’s left.

**Figure 4:**
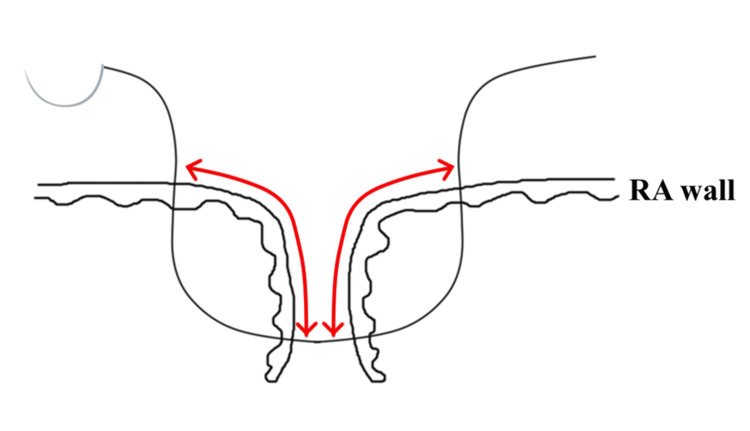
The right atrial closure The incision line was sutured with 5-0 polypropylene, ensuring inversion to achieve optimal coaptation. The red bidirectional arrows indicate the coaptation area. Image Credits: Shunya Ono. RA: right atrium

The tricuspid valve exhibited significant annular dilation, rendering repair unfeasible. TVR was performed using a 33 mm Epic Plus Mitral bioprosthetic valve (Abbott, Santa Clara, California, USA) in the same sitting. As no thrombus was observed in the LA, the heart was mobilized and exteriorized to allow closure of the left atrial appendage at its base using an AtriClip device (Atricure, West Chester, Ohio, USA). The RA was plicated by creating a 3 cm strip-like incision along the original incision line. The incision was closed in a single layer using continuous 5-0 polypropylene sutures, ensuring that the incision line was inverted during closure to improve hemostasis (Figures 3, 4). Due to the anticipated difficulty in placing a pacemaker lead after TVR, a permanent epicardial lead was sutured to the diaphragmatic surface of the right ventricle to ensure pacing capability in case a transvenous pacemaker is needed in the future. The surgery was completed successfully, with an uneventful weaning from CPB, and the patient was transferred to the ICU with minimal catecholamine support; norepinephrine was administered at a dosage of 0.05 µg/kg/min.

The patient was extubated on postoperative day (POD) 1, and a CXR showed a significant reduction in the CTR from 80% to 52% (Figures 1A, 1B). Echocardiography revealed a marked reduction in RA size from 114 × 82 mm to 55 × 47 mm (Figures 2A, 2B), with the tricuspid prosthetic valve functioning normally and no evidence of residual MR. However, the TAPSE decreased from 14.6 mm to 12.0 mm postoperatively. CT found that the lung volume had improved from 2723 cc preoperatively to 3003 cc. On POD 18, PFT revealed an improvement in VC from 1.1 L (53%) to 1.5 L (73%). Her NYHA classification improved significantly from class III to class I. Throughout the postoperative period, the patient maintained an appropriate heart rate in AF without any episodes of supraventricular tachycardia or other arrhythmias. The patient recovered well and was discharged on POD 20. At follow-up, she reported complete resolution of breathlessness, even during daily activities, and was able to move freely without limitations.

## Discussion

This case highlights the benefits of RAP combined with TVR, which led to improved respiratory function and symptom relief in a patient with severe RAE and TR. RAE, commonly associated with long-standing AF and TR, can lead to adverse outcomes such as HF and increased mortality risk [1,2].

Although LAP is more frequently reported to improve pulmonary function, similar outcomes following RAP are rarely documented [3,4]. RAP was performed in this case to address the significant RAE that was contributing to restrictive ventilatory impairment by compressing the lung volume. Preoperative imaging revealed an RA size of 114 × 82 mm, which decreased to 55 × 47 mm postoperatively. This reduction was associated with an improvement in lung volume from 2723 cc to 3003 cc and an increase in VC from 1.1 L (53%) to 1.5 L (73%). These findings suggest that the reduction in RA size contributed significantly to symptomatic relief, particularly the resolution of breathlessness.

During the closure of the RA, the thin-walled, severely dilated atrium was addressed by inverting the incision line to enhance coaptation thickness (Figure 4). This technique improves hemostasis and structural integrity, eliminating the need for additional post-closure sutures. This approach represents a significant advancement in the surgical management of RAP, particularly in cases with extreme atrial enlargement or structural weakness.

Specific RAP techniques have rarely been discussed in the literature [1]. Plication was achieved by suturing the atrial septum, excising a strip along the RA incision line, and closing the incision using the inverted suture technique. Although this procedure yielded excellent results, tailoring the surgical approach to the patient’s specific anatomy remains critical.

Concurrent TVR effectively addresses severe annular dilatation and leaflet atrophy, thereby avoiding potential repair-related complications [5]. Although TVR has historically been associated with poorer outcomes than repair [6,7], recent studies have suggested comparable long-term survival and reduced adverse events under optimized perioperative care [8].

TAPSE is a valuable parameter for assessing RV function and is widely used due to its simplicity and reproducibility [9]. In this case, a postoperative decrease in TAPSE was observed, consistent with previous reports that TAPSE often declines after cardiac surgery due to factors such as pericardiotomy and altered RV mechanics [10]. Despite the decrease in TAPSE from 14.6 mm to 12.0 mm, the patient showed no signs of right HF postoperatively. This suggests that symptomatic improvement, especially breathlessness relief, was primarily due to increased lung volume and alleviation of restrictive ventilatory impairment following the reduction in right atrial size.

Limited data on RAP restrict the generalizability of this approach. Further studies are needed to evaluate the long-term impact of atrial volume reduction on respiratory function and overall prognosis. Incorporating standardized techniques, such as the inverted closure method described here, into larger studies could provide robust evidence for their effectiveness in improving outcomes and preventing complications.

## Conclusions

This case demonstrates that RAP combined with TVR can result in significant symptomatic relief, improved pulmonary function, and a better quality of life in patients with severe RAE. The techniques employed in this study offer a reproducible and effective strategy to address severe RAE. This case highlights the importance of addressing restrictive ventilatory impairment caused by massive RAE, a major contributor to the patient's symptoms. Compared to previous studies, this report emphasizes the potential role of RAP in improving lung function through atrial volume reduction, a topic that has been less frequently discussed in the context of RAE. Furthermore, the use of the inverted suture technique during RAP, which provided both structural integrity and hemostasis, represents a notable advancement in the surgical approach for such challenging cases. Future studies could explore the efficacy of RAP in larger cohorts to validate these findings and assess its long-term impact on respiratory function and quality-of-life metrics.
